# Comparison of a Personalized Prostate Biopsy Pattern With Traditional Transrectal Prostate Biopsy: Different Cancer Detection Rate

**DOI:** 10.3389/fcell.2022.851359

**Published:** 2022-05-04

**Authors:** Xin Jiang, Sifeng Qu, Yaofeng Zhu, Shuo Wang, Haoyu Sun, Hu Guo, Benkang Shi, Shouzhen Chen

**Affiliations:** ^1^ Department of Urology, Qilu Hospital, Cheeloo College of Medicine, Shandong University, Jinan, China; ^2^ School of Medicine, Cheeloo College of Medicine, Shandong University, Jinan, China

**Keywords:** biopsy pattern, cognitive fusion-guided, prostate cancer, cancer diagnosis, detection rate

## Abstract

**Background:** In terms of prostate biopsy approaches, it is difficult to reach the ventral central region of the prostate with the traditional transrectal prostate biopsy, while with the transperineal biopsy, the tumor in the dorsolateral region of the prostate is easily missed. However, until now, no studies have investigated the biopsy accuracy in the selective application of transrectal or transperineal biopsies according to the lesion site.

**Methods:** We developed a personalized prostate biopsy pattern and the biopsy approach was selected individually according to the lesion site. We compared it with the traditional transrectal prostate biopsy method to evaluate the efficiency. Patients (*n* = 351) who underwent prostate biopsy at Qilu Hospital of Shandong University from January 2018 to October 2020 were divided into two groups, including the traditional transrectal prostate biopsy group (*n* = 236) and the personalized group (*n* = 115). The data from patients, including clinical characteristics, biopsy results, and complications, were analyzed.

**Results:** The clinical characteristics of the two groups were similar. The total detection rate of prostate cancer in the personalized group was 49.6%, which was significantly higher than 38.1% in the traditional group (*p* = 0.023). When prostate-specific antigen was <20 ng/ml, the detection rates of the two groups were 30.4 and 19.3%, respectively (*p* = 0.039). The PI-RADS was positively associated with high-grade prostate cancer in the personalized group. Patients with complications in the traditional transrectal systematic method group accounted for 6.8%, and those in the personalized group complications through the transrectal and transperineal approaches accounted for 7.1 and 4.1%, respectively. The most common complications in the transrectal group were fever and rectal bleeding, and those in the transperineal group were hematuria and urinary retention.

**Conclusion:** Compared with traditional transrectal prostate biopsy, the personalized biopsy pattern improved the detection rate of prostate cancer. The complications of the transrectal approach were much higher than those in the transperineal approach.

## Introduction

Prostate cancer is one of the most common malignant tumors in men worldwide, and it remains a major cause of cancer deaths (Caggiano et al., 2019; Kneppers et al., 2019). It is important to diagnose prostate cancer for subsequent treatment, and prostate biopsy is the crucial approach for prostate cancer diagnosis (Ried et al., 2020; Yang et al., 2020). There are two kinds of biopsy methods: transrectal and transperineal. A recent study demonstrated that the infectious complications in transperineal biopsy were lower than those in transrectal biopsy (Pradere et al., 2021). In terms of detecting accuracy, traditional transrectal prostate biopsy is difficult to get the ventral central region of the prostate, especially the urethra region, while transperineal biopsy easily misses the tumor in the dorsolateral region of the prostate (Lee et al., 1991; Luszczak et al., 2020; Wenzel et al., 2021). In recent years, multiparametric magnetic resonance imaging (mpMRI) has been widely used in prostate biopsy. Magnetic resonance imaging-targeted biopsy was better at detecting clinically significant prostate cancer than the traditional systematic biopsy.

Based on the aforementioned points, we developed a personalized prostate biopsy pattern. The suspected lesion site was determined by mpMRI examination before biopsy, and then the biopsy approach was selected individually according to the lesion site. In addition, a new technique for mpMRI-directed cognitive fusion-guided transperineal biopsy was also applied. To evaluate the efficiency of the personalized prostate biopsy pattern, the clinical information of the biopsy patients (continuous sample) from January 2018 to October 2020 in Qilu Hospital was collected. Two groups consisting of the personalized pattern and the traditional transrectal prostate biopsy were involved in our study.

## Patient and Methods

### Patient

This is a retrospective study we conducted at Qilu Hospital of Shandong University. Patients (continuous sample) who underwent prostate biopsy in our hospital from January 2018 to October 2020 were included. Excluding those who had a history of prostate cancer treatment (radiotherapy, local therapy, or endocrine therapy) before biopsy, the patients were divided into two groups (*n* = 351): the traditional transrectal prostate biopsy group (*n* = 236) and the personalized pattern group (*n* = 115).

### Information Collection

We collected demographic data of the two groups of patients, including age, serum total prostate-specific antigen (PSA), body mass index (BMI, kg/m^2^), previous biopsy, Eastern Cooperative Oncology Group (ECOG) score, and some underlying diseases such as hypertension, diabetes, and catheterization before the biopsy. Biopsy information included the method of anesthesia and pathological results and the patient’s Prostate Imaging Reporting and Data System (PI-RADS) score. For the biopsy results, we analyzed the overall detection rate of PCa. In addition, we also selected patients whose PSA was ≤20 ng/ml for further analysis.

### Biopsy Method

First, mpMRI was performed in both traditional transrectal prostate biopsy and the personalized pattern biopsy groups. The traditional transrectal prostate biopsy was performed with 12 + 1 cores (on the basis of systematic 12 cores, the remaining one core at the suspicious area shown on the MRI by cognitive fusion biopsy).

**FIGURE 1:**
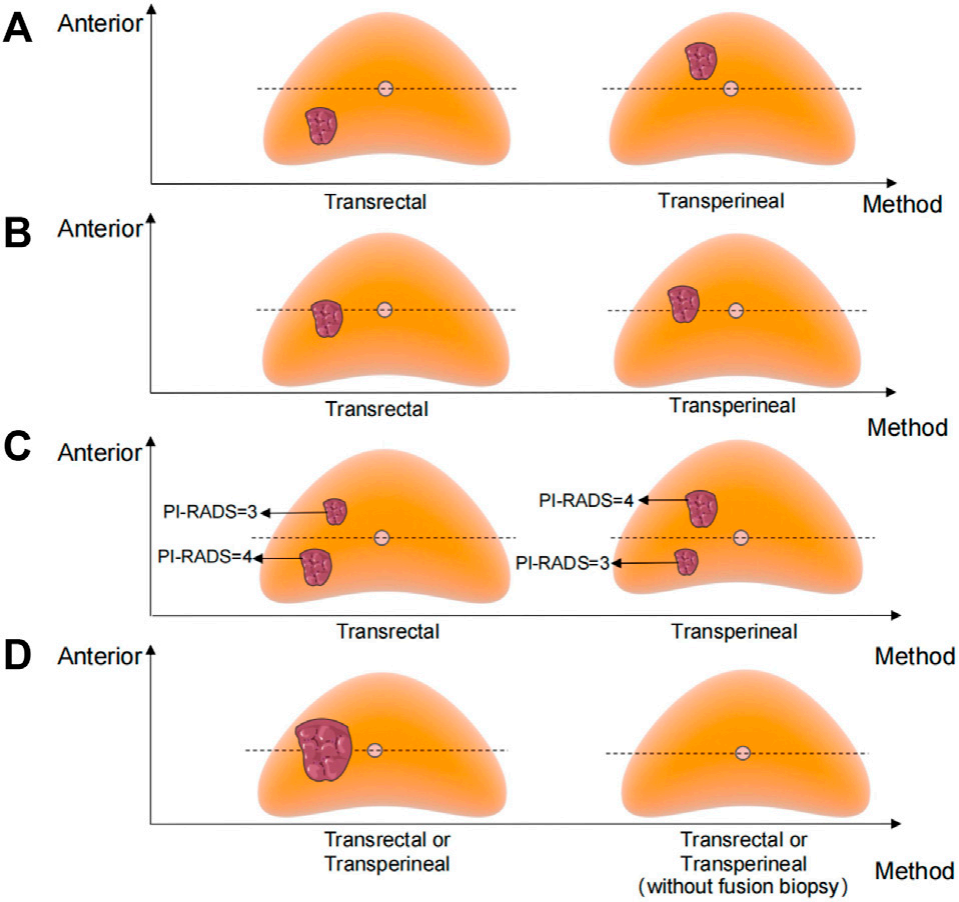
Biopsy approach of the personalized pattern. **(A)** MRI-visible prostate lesion was located in the ventral or dorsal part. **(B)** Lesion crossed the dividing line and the main part was located on the ventral or dorsal side. **(C)** More than one lesion in both ventral and dorsal parts. **(D)** Midline divided the lesion evenly or there was no obvious lesion.

The biopsy approach of the personalized pattern is shown in Figure 1. The horizontal line of the urethra divided the prostate into ventral and dorsal parts. The detailed methods are as follows: a. The transperineal approach will be selected when the MRI-visible prostate lesion was located in the ventral part. b. The transrectal approach will be selected when the lesion was located in the dorsal part. c. When the lesion crossed the dividing line and the main part was located on the ventral side, we chose the transperineal approach; otherwise, the transrectal approach. d. When there was more than one lesion in both ventral and dorsal parts, we chose the approach according to the higher PI-RADS score lesion. e If the midline divided the lesion evenly or there was no obvious lesion, either a transperineal or transrectal approach can be chosen. Most personalized transrectal approach was performed with 12 + 1 cores, same as the traditional transrectal prostate biopsy. The transperineal approach was performed with 12 + X cores (on the basis of systematic 12 cores, the remaining X cores at the suspicious area shown on the MRI).

**FIGURE 2:**
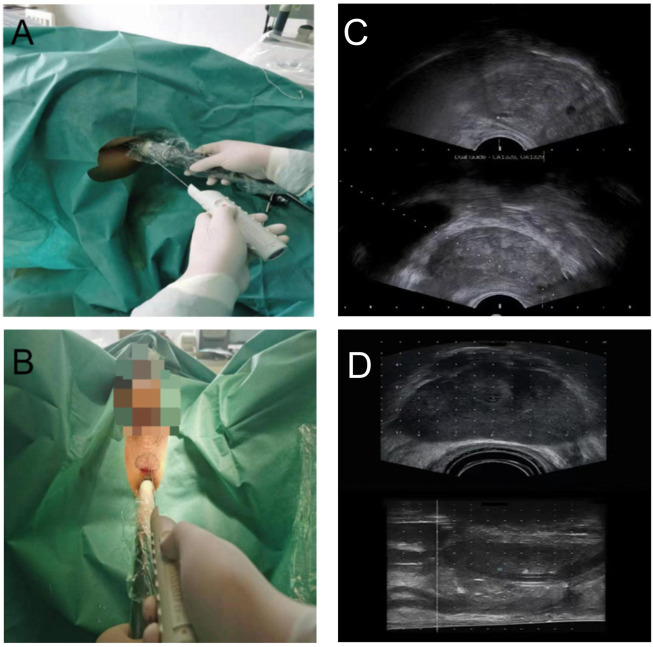
**(A)** Transrectal personalized prostate biopsy. **(B)** Transperineal personalized prostate biopsy. **(C)** Ultrasound images of transrectal biopsy. **(D)** Ultrasound images of transperineal biopsy.

Transrectal biopsy was shown in Figure 2A. Before 2 h of biopsy, 500 ml saline enema and quinolone antibiotics were used to prevent infection. We used tetracaine gel to lubricate, surface infiltration to anesthetize, and iodophor to disinfect the anus and rectum. Transrectal biopsy was performed with a B-KMEDICAL machine equipped with a probe (Type8818) for systematic biopsy and targeted biopsy at suspicious sites. The ultrasound images are shown in Figure 2C.

Transperineal biopsy is shown in Figure 2B. Before 2 h of biopsy, cefuroxime was used to prevent infection. The scrotum and testis were suspended to expose the perineal region. After disinfection with iodophor, 1% lidocaine was performed to subcutaneous infiltration anesthetize. The anus was lubricated with tetracaine gel. A B-KMEDICAL machine equipped with a probe (Type8848) was applied, and local infiltration anesthesia with 1% lidocaine was performed on both sides of the prostate apex. According to the outline of the largest cross section of the prostate from the MRI images, the outlines of the prostate and lesion were drawn in the perineal region. In the process of biopsy, the biopsy point would be adjusted by real-time ultrasound images. The ultrasound images is shown in Figure 2D.

### Statistical Analyses

Statistical analyses were performed by SPSS22.0 (Chicago, IL, United States). The clinical characteristic data were described by the median and interquartile range (IQR), and the biopsy results were described by number (n) and percentage (%). A chi-square test was used to compare the detection rate between the two groups. *p* values of both sides were taken, and *p* < 0.05 was considered to be statistically significant.

## Results

### Patient Characteristics

In this study, a total of 351 patients were involved, including 236 patients with traditional transrectal prostate biopsy and 115 patients with personalized biopsy. The clinical characteristics of all patients are shown in Table 1. The age (median and interquartile range) of patients in the personalized biopsy group and the traditional transrectal prostate biopsy group was 68 (12.25) years and 69 (11) years, respectively. The median PSA and the IQR of PSA were 16.0 (29.13) and 14.3 (24.76) ng/ml, respectively, and the median BMI and the IQR of BMI were 25.7 (3.15) and 25.4 (2.9) in each group, respectively. There was no significant difference in age, PSA, and BMI between the two groups. The number and proportion of patients in the personalized biopsy group and traditional transrectal prostate biopsy group with hypertension, diabetes, hypertension, and indwelling catheter before biopsy were 14 (12.2%) and 26 (11.0%), 30 (26.1%) and 48 (20.3%), 14 (12.2%) and 26 (11.0%), and 4 (3.5%) and 9 (3.8%), respectively. There was also no significant difference between the two groups.

**TABLE 1:** Demographic and clinical characteristics of the patients.

Characteristic	All patients	Standard transrectal biopsy	Targeted biopsy in personalized biopsy pattern
Number of patients	351	236	115
Age, years (median, IQR)	69 (12)	69 (11)	68 (12.25)
PSA, ng/ml (median, IQR)	14.8 (26.85)	14.3 (24.76)	16.0 (29.13)
BMI (median, IQR)	25.5 (3.1)	25.4 (2.9)	25.7 (3.15)
Previous biopsy, n (%)			
No.	326 (92.9)	226 (95.8)	100 (87.0)
1	24 (6.8)	10 (4.2)	14 (12.2)
≥2	1 (0.3)	0	1 (0.9)
ECOG			
0	125 (35.6)	83 (35.2)	42 (36.5)
1	124 (35.3)	81 (34.3)	43 (37.4)
2	95 (27.1)	68 (28.8)	27 (23.5)
3	7 (2.0)	4 (1.7)	3 (2.6)
Diabetes, n (%)	40 (11.4)	26 (11.0)	14 (12.2)
Hypertension, n (%)	78 (22.2)	48 (20.3)	30 (26.1)
Indwelling catheter before biopsy	13 (3.7)	9 (3.8)	4 (3.5)

### Biopsy Outcome

In general, 236 patients received traditional transrectal systematic biopsy, and 115 patients received transrectal or transperineal biopsy in the personalized biopsy group (Table 2). The number of visible targets detected by MRI was 1.2 ± 0.3, and the core number of targeted biopsies was 1 in the patients of the traditional transrectal biopsy group; the number of visible targets detected by MRI was 1.1 ± 0.2 and 1.3 ± 0.5, and the core number of the targeted biopsy was 1.2 ± 0.4 and 4.5 ± 1.7 in personalized transrectal and transperineal biopsy groups, respectively.

**TABLE 2:** Comparison of biopsy results between groups.

Characteristic	Standard transrectal biopsy	Personalized biopsy pattern	
		Transrectal	Transperineal
Number of patients	236	42	73
PI-RADS			
3	61	13	25
4	81	14	23
5	50	7	13
No. of samples on systematic biopsy	11.9 ± 0.7	11.8 ± 0.7	11.8 ± 0.5
Targeted biopsy in PI-RADS ≥3 patients			
No. of visible targets per prostate on MRI	1.2 ± 0.3	1.1 ± 0.2	1.3 ± 0.5
No. of cores on MRI-targeted biopsy	1	1.2 ± 0.4	4.5 ± 1.7

### Detection Rate of Prostate Cancer

In total, 144 (41.0%) of the 351 patients were diagnosed with prostate cancer (Table 3). The number of prostate cancer diagnosed in the traditional transrectal prostate biopsy group and the personalized biopsy group was 87 (38.1%) and 57 (49.6%), respectively. The diagnostic rate in the personalized biopsy group was significantly higher than that in the traditional transrectal prostate biopsy group (*p* < 0.05). There was no significant difference in prostate cancer, with a Gleason score of 6 between the two groups, which was possibly caused by the small sample size. There were 138 patients with a Gleason score ≥7, and the number of these patients in the two groups was 84 (36.9%) and 54 (47.0%), respectively (*p* = 0.041). As for patients with a Gleason score ≥7, the detection rate of the personalized biopsy group was significantly higher than that of the traditional transrectal prostate biopsy group.

**TABLE 3:** Comparison of cancer detection between groups.

Characteristic	All patients (*n* = 351)	Standard transrectal biopsy	Personalized biopsy pattern			*p* value
		(*n* = 236)	Overall cohort	Transrectal	Transperineal	
			(*n* = 115)	(*n* = 42)	(*n* = 73)	
Overall PCa, n (%)	144 (41.0)	87 (38.1)	57 (49.6)	24 (57.1)	33 (45.2)	0.023
Gleason score = 6, n (%)	6 (1.7)	3 (1.3)	3 (2.6)	0 (0)	3 (4.1)	0.639
Gleason score ≥7, n (%)	138 (39.3)	84 (36.9)	54 (47.0)	24 (57.1)	30 (41.1)	0.041

The comparison of the cancer detection rate between the two groups in patients with a PSA <20 ng/ml is shown in Table 4. The total number of patients diagnosed with prostate cancer was 48 (21.9%). Overall, 27 (19.3%) and 21 (30.4%) were detected in the traditional transrectal prostate biopsy group and the personalized biopsy pattern group, respectively (*p* = 0.039). The detection rate of prostate cancer in the personalized biopsy group was significantly higher than that in the traditional transrectal prostate biopsy group (*p* < 0.05). There were 43 patients with a Gleason score ≥7. Among these patients, 24(16.0%) were in the personalized biopsy group and 19(27.5%) were in the traditional transrectal prostate biopsy group (*p* = 0.046). As for patients with a Gleason score ≥7, the detection rate of the personalized biopsy group was also significantly higher than that of the traditional transrectal prostate biopsy group (*p* < 0.05).

**TABLE 4:** Comparison of cancer detection between groups in patients with PSA<20 ng/ml.

Characteristic	All patients	Standard transrectal biopsy	Personalized biopsy pattern			*p*-value
	(*n* = 219)	(*n* = 150)	Overall cohort	Transrectal	Transperineal	
			(*n* = 69)	(n = 21)	(n = 48)	
Overall PCa	48 (21.9)	27 (19.3)	21 (30.4)	6 (28.6)	15 (31.3)	0.039
n (%)						
Gleason score = 6, n (%)	5 (2.3)	3 (2.0)	2 (2.9)	0 (0)	2 (4.2)	1
Gleason score ≥7, n (%)	43 (19.6)	24 (16.0)	19 (27.5)	6 (28.6)	13 (27.1)	0.046

**FIGURE 3:**
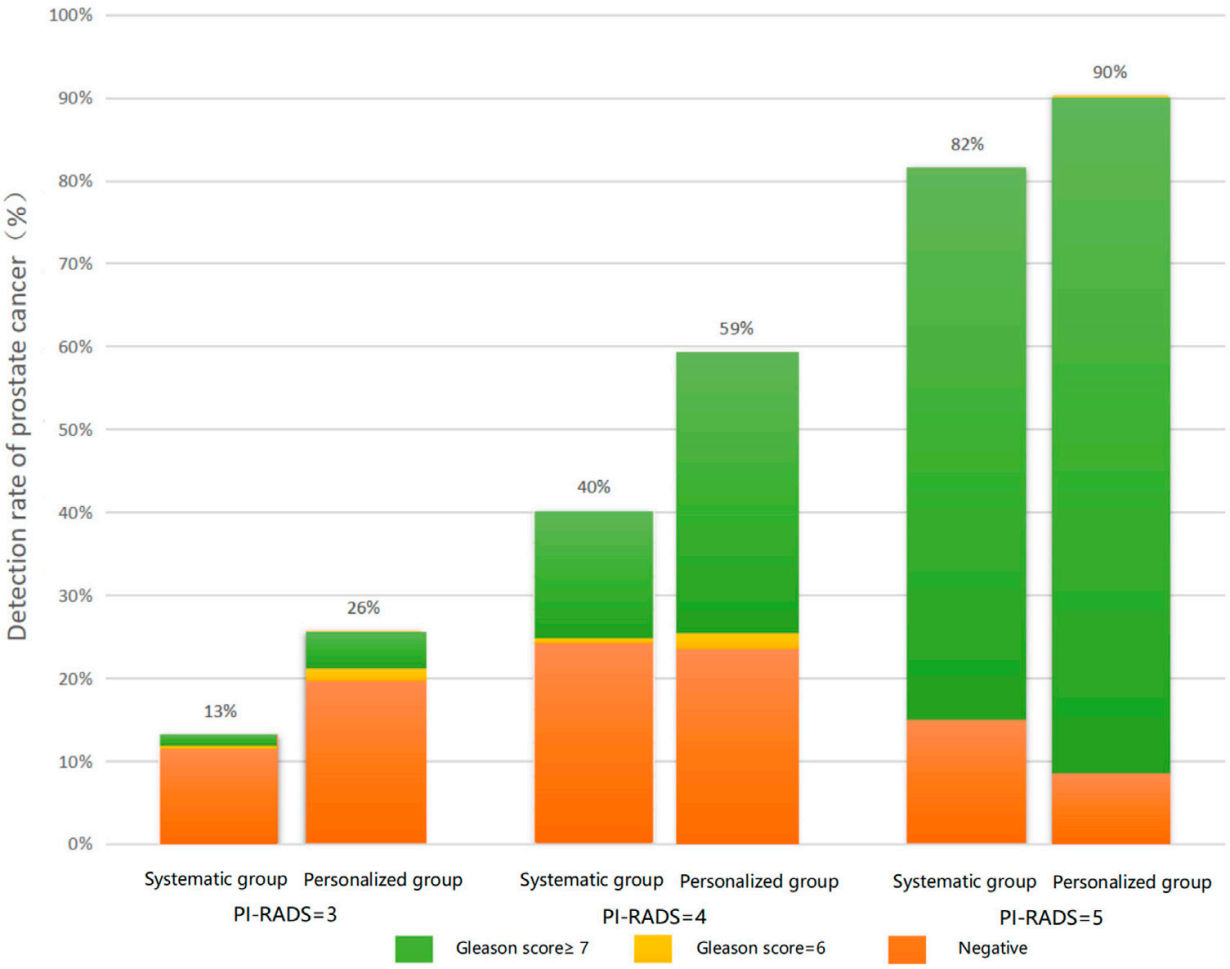
Relationship between PI-RADS, Gleason score, and the detection rate of prostate cancer in the personalized biopsy group and traditional transrectal prostate biopsy group.

The relationship between PI-RADS, Gleason score, and the detection rate of prostate cancer in each group is shown in Figure 3.

Among patients with a PI-RADS score of 3, the overall detection rate in the personalized biopsy group (26%) was significantly higher than that in the traditional transrectal prostate biopsy group (13%). The same trend was found in patients with PI-RADS scores 4 and 5, with detection rates of 59 and 40%, and 90 and 82%, respectively. Furthermore, in patients of the personalized biopsy group with PI-RADS = 3, 5% of patients were diagnosed with a Gleason score 6 and 21% of patients were diagnosed with a Gleason score ≥7. Among patients with PI-RADS = 4, 3% of patients were diagnosed with a Gleason score 6 and 56% of patients were diagnosed with a Gleason score ≥7. Among patients with PI-RADS = 5, 10% of patients were found to be non-malignant disease and 90% of patients were diagnosed with a Gleason score ≥7. It was found that the higher the PI-RADS score was, the higher Gleason score would be diagnosed with biopsy. The same was true for the traditional transrectal prostate biopsy group.

### Biopsy-Related Complications

The preoperative and postoperative complications of all enrolled patients are described as shown in Table 5. The total number of patients with complications in the traditional transrectal prostate biopsy group was 16 (6.8%). In the personalized biopsy pattern group, the total number of patients with complications in the transrectal approach and transperineal approach was 3 (7.1%) and 3 (4.1%), respectively. Most of the complications of the transrectal approach were fever and rectal bleeding, and most of the complications of the transperineal approach were hematuria and urinary retention.

**TABLE 5:** Peri- and post-procedural complications.

Number of patient complications	Standard transrectal biopsy	Targeted biopsy in personalized biopsy pattern	
	(n = 236)	Transrectal (n = 42)	Transperineal (n = 73)
22	16 (6.8)	3 (7.1)	3 (4.1)
Types of complications			
Hematuria	3	1	1
Uroschesis	5	1	1
Fever	5	1	—
Rectorrhagia	2	—	—
Urinary tract infection	1	—	—
Hematuria and uroschesis	—	—	1

### Typical Case Report

**FIGURE 4:**
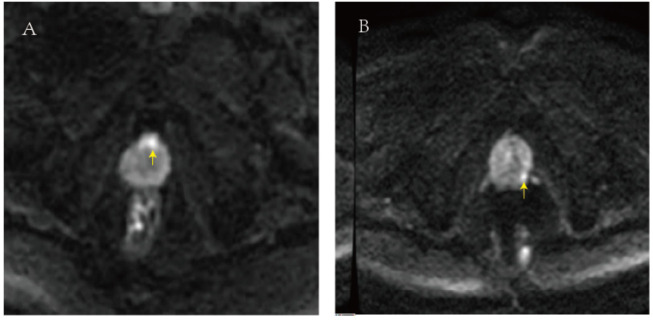
**(A)** In the image of case 1, the lesion was located on the ventral part of the prostate. **(B)** In the image of case 2, the lesion was located on the dorsal and lateral parts of the prostate.

The image of case 1 is shown in Figure 4A, and the lesion was located on the ventral part of the prostate. The patient in case 1 was 59 years old with a PSA of 13.2 ng/ml. The first-time biopsy was the transrectal route; however, no cancer was detected. The second time biopsy was the transperineal route, and the target region was prostatic adenocarcinoma with a Gleason score of 4 + 4. The image of case 2 is presented in Figure 4B, and the lesion was located on the dorsal and lateral parts of the prostate. Case 2 was a 64-year-old patient with a PSA of 9.8 ng/ml. The first-time biopsy was the transperineal route; however, no cancer was detected. The second time biopsy was the transrectal route, and the suspect region was prostatic adenocarcinoma with a Gleason score of 3 + 4. According to the two cases, the personalized biopsy pattern demonstrated noteworthy strengths. The biopsy route can be selected according to the location of the suspicious lesion in MRI to increase biopsy accuracy.

## Discussion

In our study, the cancer detection rate of the personalized biopsy group was 49.6%. In a study of perineal template-guided prostate biopsy conducted at Changhai Hospital, the cancer detection rate was 34.35% (He et al., 2019). A freehand biopsy method research showed that the cancer detection rate was 60.7% (Ristau et al., 2018). In another study that included 1,014 patients, the detection rate of cancer was 39.4% (Marra et al., 2021). We think that the difference may be caused by the different incidences of prostate cancer in different regions and countries. The incidence of prostate cancer in the Americas and Europe is significantly higher compared with that in Asia (Culp et al., 2020). In addition, the enrolled patients were of different age distribution, PSA levels, PI-RADS scores, and different biopsy methods.

There are two approaches to prostate biopsy: transperineal and transrectal. For decades, transrectal ultrasound-guided prostate core biopsy is a standard procedure for prostate cancer diagnosis. However, there has been a lot of debate on the selection of biopsy routine (Marra et al., 2019). Recent studies have reported a higher sensitivity for clinically significant prostate cancer by the transperineal approach, and this advantage is particularly obvious in the detection of tumors located in the ventral prostate (Hanna et al., 2019). This may be related to the fact that transrectal biopsy is more convenient to enter the dorsal side of the prostate, while ventral tumors are difficult to be reached. It is easy to get the ventral side and apex of the prostate by transperineal routine; however, it is easy to miss the lesions on the dorsal and lateral parts of the prostate (Schouten et al., 2017). A transrectal biopsy using the end-fire ultrasound probe could also reach the ventral side of the prostate in a previous study (Galosi et al., 2010). However, we used the biplanar side-fire probe to perform the transrectal biopsy in the present study, which was easy to miss the ventral part of the prostate. According to the aforementioned characteristics, the personalized biopsy pattern was conducted in our study. The biopsy approach can be flexibly selected according to the target region in the MRI image of the patient. In the results obtained in patients with a PSA ≤ 20 ng/ml of the personalized biopsy pattern, the detection rate of the transperineal route was 31.3%, and that of the rectal route was 28.6%. There was no significant difference between the two methods, but the detection rate was significantly higher than that of the traditional transrectal prostate biopsy (19.3%).

In terms of biopsy complications, a retrospective study of 242 patients showed that the incidence of infections was significantly higher in the transrectal group than that in the transperineal group (Huang et al., 2016). This is possibly due to the fact that the rectum is often disinfected incompletely during the transrectal biopsy, and the bacteria in the rectum could easily enter the blood or prostate, whereas the perineal skin can be disinfected thoroughly. Therefore, the infection rate was very low when the transperineal route was chosen. A systematic review also showed that transrectal biopsy had a higher incidence of infection complications, but the incidence of acute urinary retention after the biopsy was higher in the transperineal route (Pradere et al., 2021). In our study, it was found that fever and rectal bleeding were the most common transrectal complications, and hematuria and urinary retention were the most common transperineal complications (Bennett et al., 2016).

Currently, the use of mpMRI-guided targeted biopsy improved the accuracy of diagnosis. Compared with traditional systematic biopsy, MRI-targeted biopsy showed advantages in the diagnosis of clinically significant cancer. There are studies indicating that a combination of targeted and systematic biopsies can improve cancer detection rates and decrease the rate of pathologic upgrades after radical prostatectomy (Kasivisvanathan et al., 2019; Ahdoot et al., 2020). However, these technologies are expensive, and systematic biopsy is still an acceptable approach in centers that do not have the conditions to perform mpMRI. The Prostate Imaging Reporting and Data System (PI-RADS) was used in 2012 to standardize and systematize the diagnosis of MRI images of the prostate. Clinically, the system has been used to grade prostate cancer (Wegelin et al., 2017; Xiang et al., 2019). In addition, we also found that the higher the PI-RADS score, the more likely the pathological outcome with a high Gleason score.

MRI-targeted biopsy can be performed by cognitive guidance, ultrasound/magnetic resonance fusion software, or direct biopsy under the guidance of MRI (Hamid et al., 2019). The transperineal biopsy in our study was conducted by using the freehand cognitive fusion biopsy method. We innovatively delineated the outline of the prostate in the perineal region based on the maximum cross section of prostate MRI. According to the point sketched in advance and the comprehensive judgment of real-time ultrasound images during the operation, the biopsy point was selected. Compared with the current template-guided transperineal biopsy method, the freehand biopsy does not need to prepare the template but simply outlines the contour and target region in the patients perineum, which reduces the biopsy preparation time, consumable materials, and economic costs to some extent. In terms of accuracy, the freehand cognitive fusion biopsy highly depends on the operator’s judgment of the MRI image and the description of the prostate position and size, which requires a high level of experience of the operator, while the template-guided transperineal biopsy requires a relatively low level of experience due to the assistance of the template. In terms of practicability, the freehand biopsy allows the surgeon to freely change the insertion point and direction of the needle. The template-guided biopsy is limited by the position of the fixed hole in the template and cannot flexibly adjust the position and direction of the needle insertion (Mai et al., 2018; Voss et al., 2018; Maggi et al., 2021). The freehand congenital transperineal biopsy was conducted with the help of the biplane ultrasound probe consisting of the cross-sectional plane and sagittal plane in our center. We could obtain a precise location according to the combination of the cross-sectional plane and sagittal plane, which could form an accurate three-dimensional location.

As a retrospective study, there are some unavoidable limitations. We have chosen a continuous inclusion scheme to avoid selection bias. There is clearly a need for prospective, multicenter, large-scale trials to further investigate the efficiency and safety of this new personalized prostate biopsy pattern. Even so, based on our results so far, the personalized biopsy pattern was feasible and superior to the traditional transrectal prostate biopsy in terms of cancer detection.

## Conclusion

In the present study, a personalized prostate biopsy pattern was developed. The biopsy approach was selected individually according to the lesion site determined by mpMRI examination before the biopsy. A new technique for the mpMRI-directed cognitive fusion-guided transperineal biopsy was also applied. Compared with the traditional transrectal prostate biopsy, the personalized biopsy pattern improved the detection rate of prostate cancer, which was feasible and might be applied in further studies of a large number of populations in multicenter.

## Data Availability

The raw data supporting the conclusion of this article will be made available by the authors, without undue reservation.
